# The New Era of Systemic Treatment for Hepatocellular Carcinoma: From the First Line to the Optimal Sequence

**DOI:** 10.3390/curroncol30100633

**Published:** 2023-09-26

**Authors:** Maria Cerreto, Ferdinando Cardone, Lucia Cerrito, Leonardo Stella, Francesco Santopaolo, Maria Pallozzi, Antonio Gasbarrini, Francesca Romana Ponziani

**Affiliations:** 1 Liver Unit—CEMAD, Centro Malattie Apparato Digerente, Medicina Interna e Gastroenterologia, Fondazione Policlinico Universitario Gemelli IRCCS, 00168 Rome, Italy; maria.cerreto01@icatt.it (M.C.); luca.cardone27@gmail.com (F.C.); lucia.cerrito@hotmail.it (L.C.); leonardo.stella@guest.policlinicogemelli.it (L.S.); santopaolofrancesco@gmail.com (F.S.); mariapallozziucsc@gmail.com (M.P.); antonio.gasbarrini@unicatt.it (A.G.); 2 Dipartimento di Medicina e Chirurgia Traslazionale, Università Cattolica del Sacro Cuore, 00168 Rome, Italy

**Keywords:** hepatocellular carcinoma, atezolizumab, bevacizumab, sorafenib, lenvatinib, TKI, ICI

## Abstract

Hepatocellular carcinoma (HCC) represents the most common primary liver cancer and is considered a major global health problem as one of the leading causes of cancer-related death in the world. Due to the increase in life expectancy and the epidemiological growth of specific risk factors, such as metabolic dysfunction-associated steatotic liver disease (MASLD), the incidence of HCC is growing globally, and mortality rates are still high. Moreover, patients frequently present at an intermediate or advanced tumor stage, when curative treatments, such as surgical resection, liver transplantation or ablation are no longer applicable. In these cases, trans-arterial chemoembolization (TACE), trans-arterial radioembolization (TARE), and systemic therapy are the only suitable options to achieve disease control. The multi-kinase inhibitor Sorafenib has been the only systemic treatment available for unresectable advanced HCC for almost a decade, but in the last couple of years new therapeutic options have emerged. Recent advances in understanding the interactions between the tumor and its microenvironment, especially cancer immune escape, led to the advent of immunotherapy. Currently, first-line systemic treatment for HCC is represented by the combination of the immune checkpoint inhibitor (ICI) Atezolizumab plus Bevacizumab, an anti-vascular endothelial growth factor (VEGF) monoclonal antibody, but many other ICIs have been investigated, such as Nivolumab, Pembrolizumab, Durvalumab and Ipilimumab. However, the problem of second- and third-line therapies, and the correct sequence of treatments remains open and is not addressed in most studies. This explains the urge to find new systemic treatments that can improve the survival and quality of life in patients that can go beyond the first line of treatment. The aim of this paper is to offer a complete overview of the most recent innovations in systemic treatments for unresectable locally advanced and metastatic HCC, including emerging therapies, with a particular focus on treatment sequences. Moreover, we will provide an outlook on possible future approaches to patients who progress beyond first-line therapies.

## 1. Introduction

Hepatocellular carcinoma (HCC) accounts for nearly 90% of primary liver cancers and is an important cause of morbidity and mortality, as well as a leading cause of death worldwide [1]. In 2020, an estimated 905,700 people were diagnosed with liver cancer and 830,200 died, globally [2,3]. The majority of HCCs are associated with a known underlying etiology. Chronic viral hepatitis, alcohol intake and aflatoxin exposure are still the prominent causes of HCC, but tumors associated with metabolic dysfunction-associated steatotic liver disease (MASLD) are dramatically increasing, due to the global spread of predisposing factors such as obesity, diabetes and metabolic syndrome [1]. 

**Figure 1:**
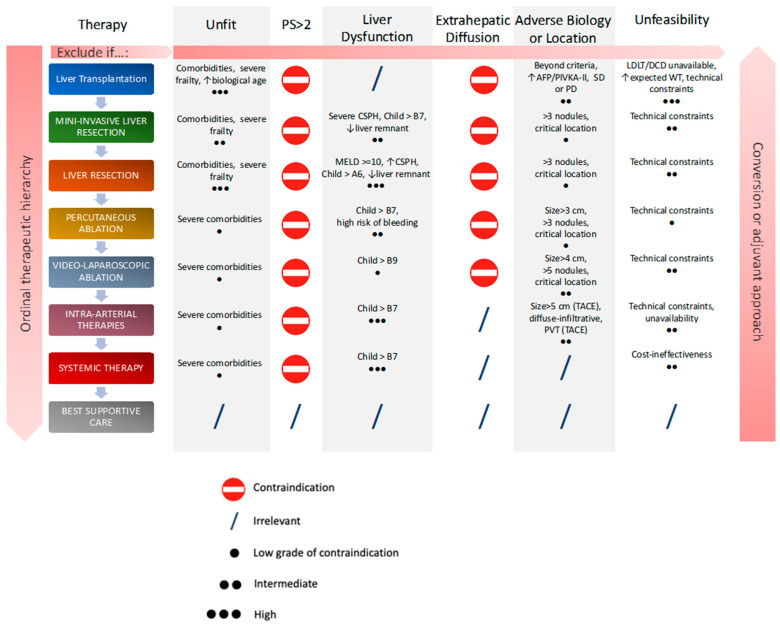
Multiparametric therapeutic hierarchy. As discussed earlier, several variables are involved in the personalized decision of the optimal therapeutic strategy. If some conditions are not met, it is possible to scale up to the treatment in the next step. Conversely, in case of therapeutic success, with the recovery of previously unmet conditions, a conversion approach can be adopted and an upgrade of the therapeutic choice can be made. The algorithm can be re-run at each decision-making point in the patient’s medical history, without any limitation, re-evaluating each factor each time to best fit the multidisciplinary decision. Abbreviations: AFP = alpha-fetoprotein; CSPH = clinically significant portal hypertension; DCD = donor after circulatory death; LDLT = living donor liver transplantation; MELD = model for end-stage liver disease; PD = progressive disease; PIVKA-II = protein induced by vitamin-K absence-II; PS = performance status; PVT = portal vein thrombosis; SD = stable disease; TACE = trans-arterial chemoembolization; WT = waiting time.

The rising incidence of HCC and the still-high mortality rate are putting increasing attention on finding new therapeutic solutions. Patients are frequently diagnosed at an intermediate or advanced stage of the disease, when locoregional treatments, such as surgical resection, liver transplantation and ablation are no longer applicable [4]. Moreover, HCC is notoriously resistant to cytotoxic chemotherapy and several trials, both for monotherapies and combinations, showed no improvement in patients’ survival [5]. Given these premises, it is important to focus our attention on advanced therapies, moving from trans-arterial chemoembolization (TACE) or radioembolization (TARE) to systemic treatments, such as target therapies and immunotherapy [4], in order to improve survival and patients’ quality of life [6]. Treatment choice relies on multiple factors, mainly patients’ fitness, critical tumor features (e.g., extra-hepatic spread, adverse biology, location) liver function and technical considerations. With remarkable improvements in the plethora of available treatments, it has become clear that we can no longer rely on treatment allocation systems based only on disease stage, but there is a need to adopt new treatment hierarchies tailored to the individual patient, with decisions guided by a multidisciplinary tumor board (Figure 1) [7].

This review is focused on the state-of-the-art of systemic therapeutic approaches to HCC and will discuss the relevance of defining a personalized strategy that should involve the following treatment lines tailored to a single patient.

## 2. First-Line Approach to HCC

### 2.1. Currently Approved First-Line Therapies

For more than a decade, the only systemic treatment with a proven efficacy on advanced HCC was Sorafenib, a multi-targeted tyrosine-kinase inhibitor (TKI) approved in 2007 [4]. Sorafenib has been the first effective systemic therapy approved for patients with advanced HCC based on the results of the SHARP trial [8], with an overall survival (OS) of 10.7 vs. 7.9 months with the placebo (HR 0.69, C.I. 0.55 to 0.87, *p* < 0.001) and a prolonged time to radiologic progression (5.5 vs. 2.8 months, *p* < 0.001). The main side effects were diarrhea, weight loss and a hand–foot skin reaction.

In 2018 Kudo et al. conducted the phase III REFLECT trial which demonstrated the non-inferiority of the TKI Lenvatinib over Sorafenib in prolonging OS [9], even if superior results were reported with regard to progression-free survival (PFS) and overall response rate (ORR), that were the secondary outcomes of the study. The main side effects of Lenvatinib were hypertension, proteinuria and diarrhea. However, the study included only patients with advanced HCC without main portal vein invasion or bile duct invasion, and with a tumor burden of less than 50% of the total liver volume [1]. 

Finally, the efficacy of Atezolizumab-Bevacizumab as a first-line treatment in non-resectable HCC has been assessed with the phase III randomized trial IMbrave150, that compared 336 patients treated with Atezolizumab-Bevacizumab and 165 patients treated with Sorafenib [10]. This study showed a statistically significant improvement in OS (67.2% (95% CI, 61.3–73.1) against 54.6% (95% CI, 45.2–64.0) at 12 months) and PFS (6.8 months (95% CI, 5.7–8.3) against 4.3 (95% CI, 4.0 to 5.6)) with Atezolizumab-Bevacizumab compared to Sorafenib [10]. In an updated analysis conducted after a median follow-up of 15.6 months, the combination therapy Atezolizumab-Bevacizumab was confirmed to obtain a better outcome both in terms of median OS (19.2 months vs. 13.4 with Sorafenib) and median PFS (6.9 months vs. 4.3) [11]. Regarding treatment safety, adverse events were reported to have a higher incidence in the group that received the combination therapy; the most common event described was hypertension, in line with the known safety profile of Bevacizumab. However, patients in the Sorafenib group experienced adverse events such as diarrhea, decreased appetite, and palmar-plantar erythrodysesthesia, leading to a poorer quality of life [12]. Similar results regarding treatment-related side effects were reported in the updated analysis. 

Recently, the phase III randomized controlled trial HIMALAYA has compared the combination of Tremelimumab (an anti-cytotoxic T-lymphocyte antigen 4 (CTLA-4) agent) plus Durvalumab (an anti-programmed death-ligand 1 (PD-L1) agent), also known as the STRIDE regimen, to Sorafenib in patients naive to systemic therapy [13]. Tremelimumab-Durvalumab was superior to Sorafenib in terms of OS (HR 0.78; 96% CI, 0.65–0.92; *p* = 0.0035) but not in PFS. At data cut-off, the median OS of the STRIDE group was 16.43 months, while that of Sorafenib group was 13.77 months; on the other hand, neither STRIDE nor Durvalumab alone extended PFS compared to Sorafenib (3.78, 3.65 and 4.07 months, respectively). Moreover, there were very few serious events of bleeding from esophageal varices during the follow-up in the STRIDE group (0.26%). Today, data from the four-year OS update are available [14], showing that the efficacy and safety of STRIDE are consistent with those of the primary analysis, in particular the OS hazard ratio for STRIDE vs. Sorafenib is 0.78 (95% CI 0.67–0.92), OS rates at 36 and 48 months are, respectively, 30.7% and 25.2% for STRIDE vs. 19.8% and 15.1% for Sorafenib. According to these data, the Durvalumab-Tremelimumab combination has been approved by the FDA [15] and could also be approved in the near future by the EMA as an alternative to Atezolizumab-Bevacizumab in the first-line setting [16].

### 2.2. How to Choose the First-Line Systemic Treatment

As previously said, Atezolizumab-Bevacizumab displayed a better OS compared to Sorafenib in patients with unresectable HCC who did not undergo previous systemic treatments; based on this, it should be preferred as the first-line approach. However, there are no randomized clinical trials that have directly compared Atezolizumab-Bevacizumab with Lenvatinib. A recent network meta-analysis demonstrated that the combination Atezolizumab-Bevacizumab granted a superior OS and PFS than Lenvatinib, Nivolumab and Sorafenib [17]. Retrospective multicenter studies reported contrasting results about Atezolizumab-Bevacizumab vs. Lenvatinib in the first-line setting; for example, some authors [18,19] found that there were no significant differences in OS in patients treated with either of these two regimens, with Atezolizumab-Bevacizumab displaying a more tolerable toxicity profile and Lenvatinib displaying a longer time to progression (TTP) in some subgroups of patients (viral etiology of HCC, Barcelona Clinic Liver Cancer (BCLC)-B stage, alpha-fetoprotein (AFP) < 400 ng/mL) [19]; other authors [19,20] found that Lenvatinib granted a better OS in patients with MASLD-related HCC, while Atezolizumab-Bevacizumab could improve survival in patients with virus-related HCC [19]. Moreover, another retrospective study [21] showed that Lenvatinib provides better OS compared to Atezolizumab-Bevacizumab in cirrhotic patients with mild deterioration of liver function (Child–Pugh class B). However, the retrospective nature of these studies cannot ensure strong evidence for changing clinical practice, and for now Atezolizumab-Bevacizumab remains the first-line therapy of choice for patients with advanced HCC.

Nevertheless, there are conditions that limit the prescription of Atezolizumab-Bevacizumab in clinical practice. For example, patients with autoimmune diseases or those that require chronic systemic immunosuppression are not suitable for ICIs [10], therefore in such cases TKIs are the therapy of choice. The slightly different toxicity profile of Lenvatinib, characterized by hypertension, diarrhea, anorexia, weight loss and proteinuria, and Sorafenib, mainly associated with diarrhea and a hand–foot skin reaction, as well as the better PFS and ORR probably linked to the higher potency of Lenvatinib, should also be considered when choosing between these two drugs. In addition, attention should be paid to patients with high-risk gastric/esophageal varices, due to the increased risk of bleeding events associated with Bevacizumab [8]. 

Moreover, given the recent approval of Durvalumab-Tremelimumab by the FDA in the first-line setting, it should be established which one is the best treatment option between STRIDE and Atezolizumab-Bevacizumab. To date, there are no randomized clinical trials performing a direct comparison, but a recent meta-analysis [22] found that Atezolizumab-Bevacizumab is not statistically superior to Durvalumab-Tremelimumab in OS (HR 0.74; 95% CI 0.52–1.06). When choosing between these two regimens, it should also be considered that STRIDE is virtually free from adverse events related to Bevacizumab, most of all bleeding risk; on the other hand, main portal vein tumor thrombosis was an exclusion criteria for the HIMALAYA trial, but not for IMbrave150, thus suggesting that Atezolizumab-Bevacizumab could be a better option for this group of patients [23].

Finally, to date Sorafenib is the only treatment that can be followed by an approved second-line therapy [24]; this point should be mentioned by the clinician when establishing the treatment outline and sharing and discussing it with the patient.

## 3. Second-Line Approach to HCC

Three possible scenarios may be responsible for the interruption of a first-line treatment: (1) intolerance (expressed in terms of drug-related adverse events), (2) radiological progression or (3) symptomatic progression of the disease [25]. In the following section, we will provide a list of drugs approved in the second-line setting for HCC and the relative available data.

### 3.1. Currently Approved Second-Line Therapies Post Sorafenib

Regorafenib, Cabozantinib and Ramucirumab are the currently approved second-line therapies in patients with preserved liver function who experience progression during Sorafenib [4,26]. 

The RESORCE phase III randomized trial compared the efficacy of Regorafenib vs. placebo as a possible second-line treatment in patients who progressed after Sorafenib [27]. Regorafenib has a similar structure to Sorafenib, but a stronger action on the VEGF pathway. The study showed an increase in the median OS from 7.8 months with placebo to 10.6 months with Regorafenib and a median PFS of 3.1 vs. 1.5 months, respectively, with an efficacy independent of the dose of Sorafenib previously taken [28]. The most common clinically relevant treatment-related adverse events were hypertension, hand–foot skin reaction, fatigue and diarrhea.

Cabozantinib, a TKI with multiple targets (such as vascular endothelial growth factor receptor 2 (VEGFR2), hepatocyte growth factor receptor (HGFR) and rearranged during transfection (RET)), proved its effectiveness in the CELESTIAL phase III trial [29] conducted on 707 patients who had previously received one or also two regimens of systemic therapy. This trial proved a significant improvement in the Cabozantinib group when compared to the placebo in terms of OS (10.2 months vs. 8.0, HR 0.76, 95% CI), PFS (5.2 vs. 1.9 months, HR 0.44, 95% CI) and ORR (4% vs. <1%). The most common adverse events in patients receiving Cabozantinib were palmar-plantar erythrodysesthesia, hypertension, increased aspartate aminotransferase (AST) levels, fatigue and diarrhea [30].

Ramucirumab is a monoclonal antibody with a great affinity for VEGFR2: the REACH 2 trial [31] proved its superiority over the placebo in terms of median OS (8.5 vs. 7.3 months) and PFS (2.8 vs. 1.6 months) for patients with AFP serum levels > 400 ng/mL [26]. In comparison with placebo, some high-grade adverse events were found to be more common in the Ramucirumab group, such as hypertension (13% vs. 2%) and hyponatremia (6% vs. 0%).

Apatinib, a TKI that targets VEGFR2, was studied in a phase III trial (AHELP) in China in the second-line setting (after chemotherapy and/or targeted therapy); this trial compared Apatinib with placebo, showing an improved OS in the Apatinib group (8.7 vs. 6.8 months), with a manageable safety profile [32]. The adverse events most commonly reported were hypertension (28%), hand–foot skin reaction (18%) and decreased platelet count (13%). The drug appeared to be promising in the Chinese population and it was therefore approved in the 2022 Chinese guidelines as a second-line systemic therapy for patients who progressed after Sorafenib [33]. However, it is still unknown if these results are generalizable worldwide.

In conclusion, based on positive randomized controlled trials, there are three drugs currently approved worldwide in the second-line setting after Sorafenib: Regorafenib, Cabozantinib and Ramucirumab [24]. A few other drugs have also been approved in the United States in second-line therapy after Sorafenib, based on the results of single-arm trials [24]. The main results are shown in Table 1.

### 3.2. Second-Line Options Post Lenvatinib

As for second-line therapies after Lenvatinib, to date there are no data available from phase III randomized clinical trials. Nevertheless, there is some evidence from retrospective studies that suggest immunotherapy could be the best choice in this setting [37]; in particular, one study [38], using a Markov model to simulate OS in patients with advanced HCC treated with different drugs, found that Nivolumab and Pembrolizumab could be interesting therapies to be administered after first-line therapy with Lenvatinib, with an OS of 27 and 25 months, respectively. However, in several countries Sorafenib is the only drug used after treatment with Lenvatinib [39] and immunotherapy does not seem to grant better OS compared to it, at least in a retrospective study [37], maybe because of the poor homogeneity of therapies in the “immunotherapy” group. Since Lenvatinib represents a cornerstone of treatment for advanced HCC today, randomized trials are needed in order to better understand the best option for second-line therapies after it.

### 3.3. Second-Line Options Post Atezolizumab-Bevacizumab

As in the case of Lenvatinib, there are no therapeutic regimens currently approved after Atezolizumab-Bevacizumab, and a scarce number of clinical trials have addressed the problem of the optimal sequence of treatments, making it difficult to provide strong recommendations [24,26]. 

A retrospective study including 36 patients showed that Lenvatinib following first-line therapy with anti PD-1/PD-L1 agents was effective, achieving a PFS of 10 months, an OS of 15.8 months (from the start of Lenvatinib) or 29.8 months (from the start of ICIs), and an ORR of 55.6% [40].

Recently, a simulation model based on available phase III randomized clinical trials has tried to identify the optimal sequential strategies based on risk/benefit [41]. This model has compared Sorafenib, Lenvatinib, Regorafenib, Cabozantinib and Ramucirumab in the second-line setting following Atezolizumab-Bevacizumab; the sequences with Lenvatinib or Sorafenib were the most effective, while the sequence with Sorafenib was the safest one [41]. A recent retrospective study compared different second-line therapies following Atezolizumab-Bevacizumab, finding statistically significant differences in OS. Patients treated with Lenvatinib or TACE achieved the best results in median OS (17.0 months and 15.9 months, respectively) even when compared with Sorafenib (median OS of 14.2 months). Other therapies were also studied, including Cabozantinib, Regorafenib, Ramucirumab and ICIs; however, no significant improvement in median OS was observed in any of these groups when compared to Sorafenib [37]. Another retrospective study conducted on 41 Asian patients showed the importance of a second-line therapy after Atezolizumab-Bevacizumab: the 30 patients who received a second systemic treatment had a longer post-first-line survival (PFLS) than those who did not (8.0 vs. 1.8 months). With regard to the drugs tested in the second-line setting, TKIs, both Sorafenib and Lenvatinib, have shown a superior efficacy compared with chemotherapy (FOLFOX) or other investigational agents; no differences were highlighted between Sorafenib and Lenvatinib in terms of PFLS (median 8.3 vs. 3.8 months, *p* = 0.258) and PFS (median 2.6 vs. 2.0 months, *p* = 0.095) [42]. These results were confirmed by Cabibbo et al. using a Markov model to compare several randomized trials of possible second-line therapies; the Atezolizumab + Bevacizumab − Lenvatinib sequence proved to be the most effective (median OS of 24.0 months) [41].

There are several ongoing studies that aim to find an evidence-based second-line therapy for patients previously treated with ICIs, and thus to understand the optimal treatment sequence; the main ones are reported in Table 2.

## 4. Current and Future Perspectives on Systemic Treatment for HCC

### 4.1. Is There an Optimal Treatment Sequence?

In the context of HCC, the choice of the treatment to be allocated should be made by an expert multidisciplinary tumor board on the basis of patients’ individual characteristics, with a case-by-case evaluation that should strive to achieve the best personalized management [43]. Parameters to be considered include tumor characteristics, liver function, presence of comorbidities and frailty, and last but not least patients’ preferences, as well as a precise assessment of the pure technical issues associated with each treatment and the relative impact they may have on patients’ quality of life [7]. Recently, we proposed a new treatment paradigm (Figure 1) in which the hierarchy of possible therapies is established on the basis of their proven efficacy, which involves all these crucial points and provides guidance for a multidisciplinary evaluation of the patient as a whole [7]. If the best treatment according to the algorithm could not be used, the choice would fall to the immediately least effective feasible treatment. This flexible multiparametric framework for HCC treatment allocation can be applied both in the first-, second- and third-line and at any subsequent evaluation of the patient [44]. 

Moreover, as shown by the right side arrow in Figure 1, a converse therapeutic hierarchy concept is an integral part of this allocation model, thanks to the new effective locoregional and systemic treatments available nowadays [7]. The conversion approach has the potential to increase the feasibility of curative therapies, making it possible to run the algorithm from bottom to top in case of good therapeutic response, leading to a tumor downstaging. The dynamic of this algorithm allows patients previously considered non-transplantable or inoperable to undergo surgery, thus providing a significant increase in survival and improving their prognosis [45]. Lenvatinib and Lenvatinib-based combinations currently represent the most promising options in a conversion therapy setting because of the high ORR observed with this agent [46,47]. However, recent studies are demonstrating the importance of other systemic treatments in this context, such as the combination of TKIs and ICIs [45,48].

Likewise, a similar rationale can be applied when considering these therapies with adjuvant intent [7]. The conversion and adjuvant approaches, which lead to an improvement in treatments’ feasibility and effectiveness, represent a promising opportunity and should be part of every multidisciplinary discussion on HCC treatment allocation.

The issue of the treatment of advanced HCC has become very difficult recently. Over the past two decades, we have seen significant improvements and technical innovations in the treatment of advanced HCC, thus entering the era of sequential therapy. The approval of new agents has obviously increased the difficulty in managing the treatment strategy, also considering the very narrow number of drugs approved as second-line options. 

The results of the IMbrave150 trial led to the preferential use of the Atezolizumab-Bevacizumab combination in first-line. However, a scarcity of evidence addresses the problem of the optimal sequence of treatments after this new first-line combination [24,26], and Sorafenib currently remains the only treatment that can be followed by an approved second-line therapy.

Given our current knowledge on the treatment of advanced HCC, it seems reasonable to consider the use of TKIs, namely Sorafenib, Lenvatinib, Cabozantinib or Regorafenib, after the failure of Atezolizumab-Bevacizumab [49]. In particular, some experts consider it possible to use all of these drugs in the second-line setting, while others believe that only Sorafenib and Lenvatinib should be used, reserving Regorafenib, Cabozantinib and Ramucirumab as third-lines [50,51,52]. The rationale for this hypothesis lies in the fact that these molecular-targeted agents with multi-kinase inhibitory activity seem to induce the release of cancer antigens thus prolonging the residual effect of anti-PD-1/PD-L1 drugs after treatment failure [52]. Nonetheless, to date there is no strong evidence supporting these suggestions, which are based only on expert opinion and real-world data, with a low quality of evidence and a weak strength of recommendation [49]. Promising results have been obtained regarding the use of Sorafenib and Lenvatinib after Atezolizumab-Bevacizumab, but further clinical trials are needed that may open other pathways to sequential therapy for HCC [37,41,42].

Several ongoing trials are investigating the use of ICIs after progression with Atezolizumab-Bevacizumab, opening an interesting new avenue for HCC sequential treatment. Promising results have been obtained for Nivolumab (median OS 24 months) [41] or for Nivolumab-Ipilimumab (median OS 10.9 months in a retrospective real-world study) [53], but it is hoped that new randomized trials in the future will provide even more encouraging results, taking into account other combinations initially tested in the first-line setting, such as Tremelimumab-Durvalumab. As growing evidence supports the use of combination therapies, due to the synergistic role that can be played by drugs acting on different pathways, it is also reasonable to speculate on the prominent role that ICIs plus TKIs or anti-VEGF combinations may play in the second-line treatment of advanced HCC. As mentioned in Table 2, there are several ongoing trials investigating the efficacy of these possible combinations. In the case of patients treated in the first-line setting with Atezolizumab-Bevacizumab in whom disease progression has occurred, one might consider adding a TKI while continuing with Atezolizumab-Bevacizumab or replacing it with a similar one (e.g., Durvalumab-Ramucirumab). A phase Ia/b trial (JVDJ) evaluated Durvalumab plus Ramucirumab safety and efficacy in different cancers including HCC as a first-line approach, but it is likely that this as well as other drug combinations will be tested in the second-line setting [54]. Another interesting phase Ib/II study [55] found that the association of Tiragolumab (an ICI that targets TIGIT (T cell immunoglobulin and ITIM domain), an inhibitory immune checkpoint that can be found on activated T cells and NK cells) with Atezolizumab plus Bevacizumab in the first-line setting can provide better PFS and ORR compared to Atezolizumab-Bevacizumab, but further studies are needed to confirm these results and possibly change clinical practice. Equally interesting would be the evaluation of the use of triple therapies combining a TKI with two ICIs acting on different receptors (i.e., an anti-PD-1/anti-PD-L1 with an anti-CTLA-4) or with an ICI and an anti-VEGF. 

Nevertheless, several ongoing trials are investigating the role of systemic drugs, in particular ICIs, in association with curative or locoregional treatments. The rationale behind their use in combination lies in the complementary and synergistic mechanism of action: after causing direct damage to tumor cells with locoregional treatments, thus exposing new tumor antigens, ICIs can stimulate and potentiate the immune response against them [56]. A few trials deserve to be mentioned in this regard. The first one showing positive results in the adjuvant setting is the IMbrave050, an open-label, phase III randomized study comparing Atezolizumab-Bevacizumab for 12 months or 17 cycles with active surveillance in patients at high risk of disease recurrence following liver resection or ablation. The interim analysis reported that recurrence-free survival was better in the treatment group (HR 0.72, 95% CI: 0.56–0.93; *p*-value = 0.012) with a 33% lower recurrence rate, and that was consistent across clinical subgroups [57]. The TRIPLET phase II trial (NCT04191889) aims to evaluate the efficacy and safety profile of the combination of hepatic arterial infusion chemotherapy with mFOLFOX27, a targeted drug (Apatinib), and an anti-PD-1 antibody (Camrelizumab) in patients with stage C HCC who did not receive previous systemic treatment [58]. A pilot trial (NCT02821754), on the other hand, is currently studying the potential effects of the combination Durvalumab plus Tremelimumab plus radiofrequency ablation/cryoablation/TACE in those patients who had a previous failure or intolerance with first-line Sorafenib [59]. An ongoing trial (NCT04220944) conducted on 45 participants with unresectable HCC aims to evaluate the efficacy of percutaneous microwave ablation with simultaneous TACE plus Sintilimab (anti-PD-1), but no results are available yet [60]. A single-arm phase II clinical study (NCT04945720) has been designed with the purpose of establishing the OS, efficacy and safety of the combination of a FOLFOX hepatic arterial infusion with anti-PD-L1 immunotherapy (Durvalumab); so far 30 patients with advanced HCC with portal vein thrombosis have been enrolled [61]. Another phase II trial (NCT03937830) is currently studying the use of a combined treatment with Durvalumab, Bevacizumab, Tremelimumab and TACE in subjects with advanced HCC or biliary tract carcinoma; a second group treated only with Durvalumab, Bevacizumab and Tremelimumab has been formed to compare the 6-month PFS [62]. As a final remark, the DEMAND trial protocol (NCT04224636) is currently studying the efficacy of up-front Atezolizumab-Bevacizumab followed by on-demand selective TACE and of a synchronous treatment with Atezolizumab-Bevacizumab and TACE [63]. The dual-agent Atezolizumab-Bevacizumab also proved effective and safe when combined with Y-90 TARE in a case report of a patient with advanced HCC and portal vein tumor thrombosis [64]; an ongoing phase II clinical trial (NCT04541173) is addressing this combination therapy in comparison with TARE alone [65]. These promising results should make us realize the importance of associations between locoregional treatments and systemic therapies, and further clinical trials are essential. 

Less promising seems to be the association between locoregional treatments and TKIs. Kudo et al., in the 2019 TACTICS trial, demonstrated significant superiority in median PFS of the TACE plus Sorafenib combination over TACE alone (25.2 vs. 13.5 months); secondary efficacy outcomes, namely time to untreatable progression and median time to progression, were also significantly longer in the TACE plus Sorafenib group than in the TACE alone group (26.7 vs. 20.6 months and 26.7 vs. 16.4 months, respectively) [66]. On the contrary, other studies have not demonstrated a statistically significant improvement in survival with combination therapies, neither when using Sorafenib plus TACE [67] nor when using Sorafenib plus radioembolization [68]. Recently, the LAUNCH trial compared 170 patients treated with Lenvatinib plus TACE and 168 patients treated with Lenvatinib alone after disease recurrence after surgery; after a median follow-up of 17.0 months, they reported a significantly longer median OS (17.8 vs. 11.5 months) and PFS (10.6 vs. 6.4 months) in the combination group than in the Lenvatinib alone group [69]. An interesting ongoing trial (NCT05220020) aims to compare the synchronous use of Lenvatinib and TACE with their sequential use (patients with progression after TACE treatment are sequentially treated with Lenvatinib); the two-year OS rate of the two groups will probably give us interesting information on the use of this combination in advanced HCC [61].

As a final remark, while ICIs alone or in combination with other antiangiogenic agents represent the more promising perspectives in the clinical approach to advanced HCC, other immunotherapeutic resources are currently under investigation. Even if we are still far from their use in clinical practice, in the future a relevant role could be played by adoptive cell transfer, as chimeric antigen receptor T cells (CAR-T cells) [56,58], therapeutic vaccines [59], dendritic cell therapies and therapeutic viruses [70].

### 4.2. How to Choose the Most Feasible Treatment

Many factors must be taken into consideration when deciding whether a patient is a candidate for systemic treatment and which therapy is most appropriate. Beyond the obvious evaluation of drug efficacy (in terms of OS and PFS), it will be crucial to consider the patient’s overall clinical assessment (fitness), the tumor burden, the toxicity profile and adjust the decision based on individual preferences and expected post-treatment quality of life.

**Figure 2:**
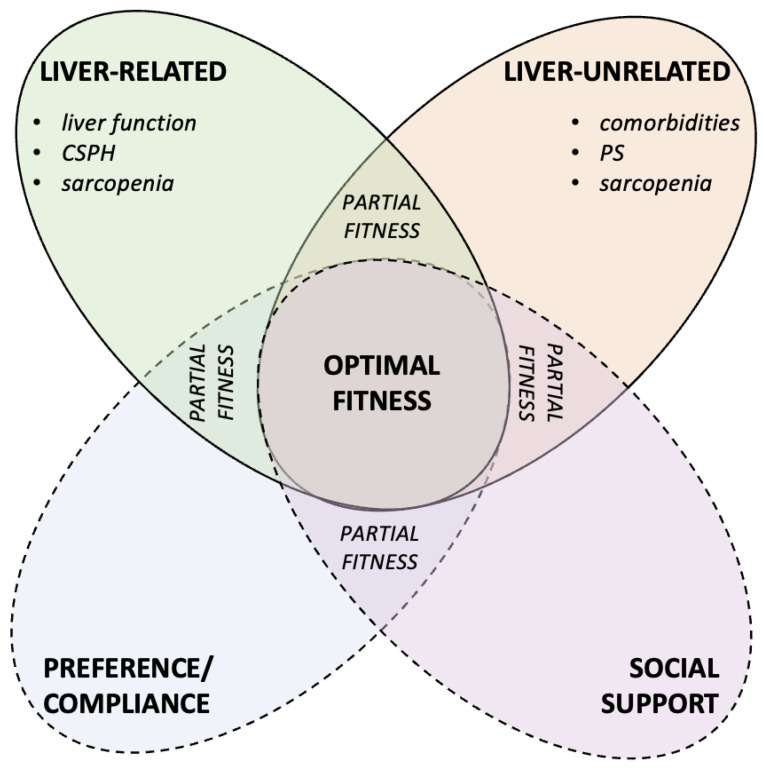
Integrated view of the patient’s fitness. Many factors are involved in the overall assessment of fitness. When liver-related and liver-unrelated variables meet the optimal requirements for the specific treatment, including patient preference/compliance and adequate support from the surrounding family/social environment, this is the optimal setting for implementing a therapeutic strategy. However, these conditions are not always realized in every patient; when the prerequisite of fitness is partially achieved, the possibility of pursuing the hypothesized course of treatment must be decided on a case-by-case basis. Abbreviations: CSPH = clinically significant portal hypertension; PS = performance status.

Major determinants in patient’s clinical evaluation include performance status and general fitness, which in patients with chronic liver disease and HCC includes liver functional reserve, portal hypertension, comorbidities, preference/compliance and social support (Figure 2) [42]. 

Phase III trials focusing on systemic treatments for HCC usually include patients with ECOG performance status (PS) 0–1; a more compromised PS is currently considered a relative contraindication to systemic therapies [71]. However, as regards PS a separate discussion should be made in the setting of HCC. In fact, it is difficult to discern between symptoms that may be related to cirrhosis or to the tumor per se, as asthenia and weight loss, in addition to limitations in the individual’s autonomy, can often be attributed to both conditions. In addition, sarcopenia, which is considered a marker of frailty, is an independent negative predictor for systemic treatment sustainability and, consequently, efficacy [51], and certain classes of drugs can worse this condition. In fact, many TKIs act indirectly on the protein kinase B (AKT)/mammalian target of rapamycin (mTOR) pathway, causing a reduction in muscle cell proliferation, protein synthesis and muscle fiber growth [72]. Consequently, it is necessary to prescribe TKIs, especially Sorafenib and Lenvatinib [73,74], with particular care in patients who are already malnourished and have sarcopenia due to HCC, and continuous patient monitoring is required to highlight any side effects of the drug [72]. Nutritional support should also accompany the patient throughout the treatment journey.

Therefore, beyond PS or sarcopenia alone, the general concept of “fitness” is a more reliable measure to be considered, as it involves a multiparametric assessment of individuals with chronic liver disease in each specific case. 

Liver function is also plays a key player in the management of systemic therapies for HCC, since it is associated with treatment duration and mortality [75,76,77]. The Child–Pugh score surely represents one of the main parameters to consider when deciding if a patient is suitable for a second-line systemic approach. Various clinical studies have shown that a good liver reserve after first-line treatment (Child–Pugh class A) is associated with a significant increase in survival [49]. On the other hand, a poor liver function after a first-line treatment failure usually undermines prognosis, precluding the possibility of a subsequent therapy [42]. However, patients with an impaired liver function (Child–Pugh B or C) tend to be excluded from clinical trials, making it difficult to provide an evidence-based treatment allocation [78]. In a meta-analysis addressing the use of Sorafenib in Child–Pugh B patients with advanced HCC, the median OS result was significantly worse than that of patients with a good liver function (4.6 vs. 8.8 months) [79]. Similar results have been obtained for Lenvatinib in a multicenter cohort study: 108 patients with Child–Pugh A, 27 with Child–Pugh B and 2 with Child–Pugh C were treated and the median OS was 12.5 months in those with preserved liver function vs. 5.6 months in those with decompensated liver function [80]. Regarding ICIs, Nivolumab as a monotherapy has been studied in cohort 5 of the CheckMate 040 trial, in patients with advanced HCC and Child–Pugh B: it demonstrated clinical activity (median OS of 7.6 months and 55% of response rate), with a favorable safety, thus suggesting it could be a potential treatment option for patients with Child–Pugh B liver function [81].

D’Alessio et al. conducted a real world study on 202 patients with advanced HCC treated with Atezolizumab-Bevacizumab. No significant difference was observed between Child–Pugh A and Child–Pugh B patients in terms of treatment-related adverse events of any grade. Comparing the two groups of patients, the median OS was 16.8 months (95% CI, 14.1–23.9) in those with Child–Pugh A and 6.7 months (95% CI, 4.3–15.6) in those with Child–Pugh B, while the median PFS was 7.6 months (95% CI, 6.2–8.9) vs. 3.4 months (95% CI, 2.6–4.2). On the other hand, the ORR was comparable across Child–Pugh classes, at 26% in Child–Pugh A and 21% in Child–Pugh B. Therefore, Atezolizumab-Bevacizumab proved to be well tolerated and effective even in Child–Pugh B patients, thus suggesting that this combination could be safely administered even beyond the strict inclusion criteria of the IMbrave150 study [82].

To complete the discussion on the use of the four first-line therapies approved to date for advanced HCC in patients with poor liver function, it is necessary to mention the Tremelimumab-Durvalumab combination in this class of patients. Vogel et al. [83] recently presented a trial on the use of the STRIDE regimen, Durvalumab alone or Sorafenib in patients with advanced HCC, irrespective of baseline albumin-bilirubin (ALBI) grade. Patients were divided according to their mild (ALBI grade 1) or moderate-to-severe (ALBI grade 2/3) impairment in liver function. OS hazard ratios (HRs) for STRIDE vs. sorafenib in the ALBI 1 (0.79; 95% CI, 0.62–1.01) and ALBI 2/3 (0.83; 95% CI, 0.65–1.05) groups were generally consistent with the full analysis set (0.78; 96% CI, 0.65–0.93); similar results were obtained for Durvalumab alone vs. Sorafenib (OS HRs in the ALBI grade 1 (0.91; 95% CI, 0.71–1.15) and ALBI grade 2/3 (0.87; 95% CI, 0.69–1.09) groups were consistent with the full analysis set (0.86; 96% CI, 0.73–1.03). Moreover, STRIDE and Durvalumab showed a higher ORR, shorter TTR (time-to-response) and longer DoR (duration of response) than Sorafenib in both ALBI subgroups. The safety profile in both ALBI groups was similar to the safety analysis set. As a final remark, both ALBI and Child–Pugh scores remained stable during STRIDE/Durvalumab treatment. These findings support the use of the STRIDE regimen as a possible treatment option in patients with advanced HCC, regardless of baseline ALBI grade [84].

In conclusion, both TKIs and ICIs, with the possible addition of an anti-VEGF, could be taken into account as possible options in selected patients with Child–Pugh B advanced HCC. On the other hand, in patients with severe liver function impairment (Child–Pugh C), systemic treatment has not demonstrated a benefit in either OS or PFS and is therefore contraindicated, even in the face of the unwieldy safety profile [50]. For patients with clinically significant portal hypertension (usually not included in the scores and management algorithms that are used routinely in clinical practice), and in particular patients with varices, prophylaxis with non-selective beta-blockers or endoscopic band ligation is necessary before taking into consideration a systemic treatment.

While age is no longer considered a contraindication to systemic treatments, patient’s comorbidities are instead a crucial point; for example, cardiovascular disease is a possible contraindication to anti-VEGF or TKI treatments because of their known risk profile with regard to hypertension and cardiovascular events [7]. Of note, the prevalence of cardiovascular diseases in patients with advanced HCC is very high, thus making an in-depth study of the best systemic therapies for this fragile population imperative. However, patients with severe cardiovascular diseases or with recent acute events are usually excluded from clinical trials testing new systemic treatments for HCC, which means that strong recommendations are lacking [78]. Given the safety profile of the different drugs and based on the recommendation of cardiology societies [85], a thorough baseline assessment of cardiac reserve is mandatory in patients candidate to anti-VEGF agents and close monitoring during and after treatment is essential; nevertheless, anti-VEGFs should be cautiously administered in patients at high–very high cardiovascular risk, and ICI-alone regimens should be considered as the preferred treatment in this class of patients, but specific data are still missing.

As previously said, autoimmune diseases or conditions that require chronic systemic immunosuppression are, on the other hand, an absolute contraindication for ICIs. 

Thus, handling not only the tumor but also the underlying liver disease and the possible episodes of decompensation is essential, in order to both avoid systemic treatment interruption due to worsening liver function and treat those patients in whom systemic therapy cannot be administered due to a transitory but recoverable impairment of liver function [43].

Several parameters should be considered when assessing tumor stage and aggressiveness; among these negative prognostic factors the most important are tumor burden, AFP serum levels, extrahepatic diffusion or vascular/biliary tree invasion, and critical tumor location [7]. Not in all phase II/III studies, however, have these elements been considered. Nevertheless, they must be carefully evaluated before proposing a systemic treatment to the patient; for example, portal vein invasion has been associated with a higher bleeding risk due to portal hypertension [86], and should lead to closer re-evaluation of portal hypertension for the risk of rapidly developing esophago-gastric varices.

It should be highlighted that the etiology of HCC seems to influence the clinical response to ICIs [87]. A meta-analysis involving 1656 patients from three major trials (IMBrave150, KEYNOTE-240 and CHECKMATE-459) was performed in order to underline the differences in OS after immunotherapy according to the underlying etiology of HCC [88]. Results showed a significant improvement only in those patients with viral HCC (HR: 0.64; 95% CI: 0.48–0.94), while no survival benefits were reported for other etiologies. On the contrary, ORR and PFS were similar beyond the cause of HCC. However, some limits of this meta-analysis should be emphasized. Firstly, the seemingly contradictory results regarding OS, ORR and PFS could be related to the heterogeneity of the non-viral population [89]; moreover, the inferior outcomes observed for non-viral HCC patients result from a retrospective analysis. Therefore, further trials with patients’ stratification are needed so that differentiated treatments according to the underlying etiology of HCC can be applied in clinical practice.

**Figure 3:**
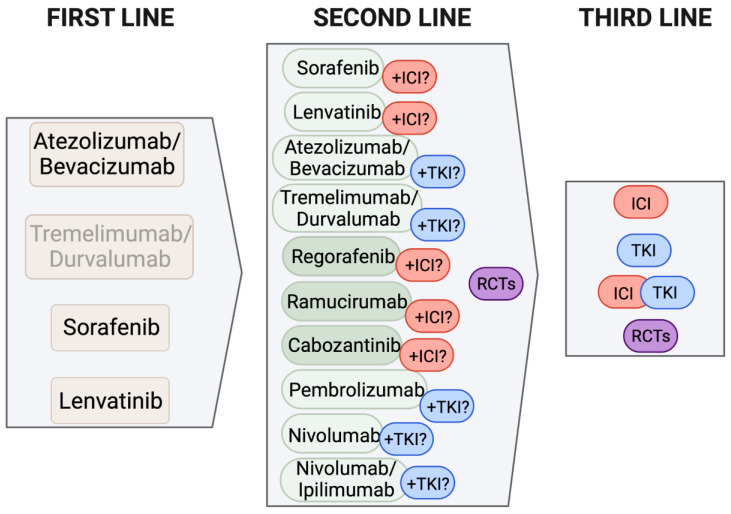
The ideal personalized sequence of approved therapies. Approved first-line regimens can be followed by any of the available TKIs or ICIs, depending on the reason for treatment discontinuation (toxicity, progression), tolerability profile, manageability (oral or infusional), and with the possibility of combining multiple agents of different classes/with different molecular targets, to achieve the best efficacy outcome. Abbreviations: ICI = immune checkpoint inhibitor; TKI = tyrosine-kinase inhibitor; RCT = randomized controlled trial. Figure created using BioRender.com.

The tolerability and toxicity profile of the different classes of drugs should also be taken into consideration [70]. The main treatment-related adverse events of TKIs include a hand–foot skin reaction, diarrhea, fatigue and hypertension, while VEGF-inhibitors are frequently the cause of hypertension, cardiovascular toxicity, proteinuria and bleeding; the use of ICIs can lead to the occurrence of immune-mediated reactions, potentially involving any organ [24]. A special mention, however, should be made for all lines of treatment after the first. In order to choose the best drug following the first-line, the reason why the first-line failed and the presence of contraindications for certain classes of drugs should be considered. For example, if the patient had an immune-related adverse event that led to treatment discontinuation of Atezolizumab-Bevacizumab, only TKIs should be considered as second-line options; on the other hand, if the reason for discontinuation was tumor progression or a Bevacizumab-related adverse event, other ICIs, TKIs or Ramucirumab can be chosen as subsequent lines. Similarly, if a patient was treated in the first line with Sorafenib or Lenvatinib because of contraindications to ICIs, other TKIs or Ramucirumab can be suitable as second-line options [43]. Finally, local availability and refundability of the drugs must be taken into account for the final decision. A hypothetical outline of the treatment decision pathway is shown in Figure 3.

All the above information must be taken into account for a tailored treatment choice, and to maintain a balance between improving survival and preserving the patient’s ability to work or perform daily activities, in some cases, and in other ones to adequately inform caregivers and prevent excessive decline in the quality of life. Finally, in establishing the correct treatment sequence, the availability of approved second- or third-line therapies should be clearly explained to the patient, to delineate from the beginning the track to be followed in the therapeutic pathway.

## 5. Conclusions

The systemic treatment of HCC underwent profound changes in recent years, mostly thanks to the growing use of ICIs. Based on currently available data, Atezolizumab-Bevacizumab represents the preferable first-line systemic treatment for unresectable HCC; Sorafenib is now the old glory of TKIs, but is still available as a first-line option along with Lenvatinib. Beyond the great efforts toward the research of new first-line therapies, such as the Durvalumab-Tremelimumab combination that will flank Atezolizumab-Bevacizumab in the first line, unfortunately, research on the second-line scenario is not moving as fast. To date, Sorafenib is the only drug that can be followed by second-line agents, the use of which is based on strong evidence. The FDA also approved other therapies in the second-line setting following Sorafenib, i.e., Pembrolizumab, Nivolumab and the combination of Nivolumab plus Ipilimumab, but with a weaker background. Unfortunately, there are no therapies approved in the second-line setting for patients previously treated with Atezolizumab-Bevacizumab; some experts believe that TKIs could be used, nonetheless there is no strong evidence supporting these statements. While combination therapies certainly represent one of the most promising prospects, CAR-T cells, therapeutic vaccines and therapeutic viruses may also have a promising future application in this scenario.

## Figures and Tables

**Table 1:** Drugs approved by the FDA as second-line therapy after Sorafenib, based on single-arm trials.

Drug	Mechanism of Action	Trial	Number of Enrolled Patients	Important Exclusion Criteria	Results	Approval
Pembrolizumab	anti PD-1	KEYNOTE-224, phase II, non-randomized [34]	156	Variceal bleeding (esophageal or gastric) or encephalopathy within the previous 6 months, clinically apparent ascites, invasion of main portal vein or inferior vena cava	ORR 18.3%, DCR 61.5%	Approved by the FDA after progression on Sorafenib
Nivolumab	anti PD-1	CheckMate 040, phase I-II, non-randomized [35]	262	Any prior or current clinically significant ascites, any history of hepatic encephalopathy	ORR 20%, DCR 64%	Approved by the FDA after progression on Sorafenib
Nivolumab + Ipilimumab	anti PD-1 + anti CTLA-4	CheckMate 040, phase I-II, randomized 1:1:1, no control group [36]	148	Any prior or current clinically significant ascites, any history of hepatic encephalopathy	ORR 32%, DCR 54%	Approved by the FDA after progression on Sorafenib

Abbreviations: PD-1 = programmed death 1; ORR = overall response rate; DCR = disease control rate; FDA = Food and Drug Administration; and CTLA-4 = cytotoxic T-lymphocyte antigen 4.

**Table 2:** Ongoing studies for second-line therapies after ICIs.

Drug	Mechanism of Action	Previous First-Line	Trial	Number of Enrolled Patients	Main Exclusion Criteria
Atezolizumab + Lenvatinib/Sorafenib vs. Lenvatinib/Sorafenib	anti PD-L1 + TKI vs. TKI	Atezolizumab-Bevacizumab	IMbrave 251, phase III, randomized 1:1, controlled, NCT04770896	554 (recruiting)	History of HE, PS ≥ 2, Child–Pugh class worse than A
Atezolizumab + Lenvatinib/Cabozantinib vs. Lenvatinib/Cabozantinib	anti PD-L1 + TKI vs. TKI	Atezolizumab-Bevacizumab	ACCRU-GI-2008, phase II, randomized 2:1, controlled, NCT05168163	122 (recruiting)	Known co-infection with HBV and HCV, untreated or incompletely treated esophageal/gastric varices at high risk for bleeding, PS ≥ 2, Child–Pugh class worse than A
Regorafenib + Pembrolizumab	TKI + anti PD-1	anti PD-1/PD-L1	Keynote B70, phase II, non-randomized, NCT04696055	95	Untreated esophageal varices at risk of bleeding, active HBV/HCV co-infection
Cabozantinib	TKI	anti PD-1/CTLA-4/PD-1 + CTLA-4	HCC063, phase II, non-randomized, NCT04588051	20	PS ≥ 3, Child–Pugh class worse than A, concomitant anticoagulation, clinically significant bleeding risk, untreated or incompletely treated varices at high risk for bleeding, moderate or severe ascites
Regorafenib + Nivolumab	TKI + anti PD-1	Sorafenib or Atezolizumab-Bevacizumab	GOING, phase I-II, non-randomized, NCT04170556	78	PS ≥ 2, Child–Pugh class worse than A, clinically meaningful variceal bleeding within the previous 3 months, clinically meaningful ascites, history of HE within the previous 12 months or requirement for medications to prevent or control HE, active HBV/HCV co-infection, concomitant anticoagulation

Abbreviations: PD-L1 = programmed death-ligand 1; TKI = tyrosine-kinase inhibitor; PS = performance status; HE = hepatic encephalopathy; HBV = hepatitis B virus; HCV = hepatitis C virus; PD-1 = programmed death 1; CTLA-4 = cytotoxic T-lymphocyte antigen 4.
